# Special Issue “Anticancer Drugs 2021”

**DOI:** 10.3390/ph15040479

**Published:** 2022-04-14

**Authors:** Mary J. Meegan, Niamh M. O’Boyle

**Affiliations:** School of Pharmacy and Pharmaceutical Sciences, Trinity Biomedical Sciences Institute, Trinity College Dublin, 152-160 Pearse Street, 2 DO2R590 Dublin, Ireland

This Special Issue of *Pharmaceuticals* is devoted to significant advances achieved in the field of Anticancer Drugs in 2021. Recent findings and trends in the design, synthesis and mechanism of action and therapeutic applications of anticancer drugs are presented. These research studies demonstrate the relevance of medicinal chemistry and the pharmaceutical sciences in cancer research. The research illustrates the exciting opportunities that contemporary drug design offers for the discovery of new therapies and diagnostics for cancer and offers perspectives on the future directions of anticancer therapeutics. Resistance to anticancer drugs has become a major threat to the success of chemotherapeutic agents, and therefore the discovery and development of new anticancer drugs for clinical use is extremely challenging. Investigations into anticancer drugs covers a vast area of research and includes natural products, design and synthesis of new molecular entities, molecular modelling, computational techniques and development of molecular and biochemical tests. Heterocycles are well represented in this collection with the inclusion of novel compounds targeting kinases, tubulin, thymidylate synthase, histone deacetylase, HER2 and apoptosis-related proteins. Original research into amelioration of toxicity associated with current chemotherapeutics and resistance is also featured.

In 2020, the FDA approved 18 new cancer drugs, including the HER2-directed margetuximab, sacituzumab govitecan [a TROP2-targeted antibody–drug conjugate (ADC) for triple-negative breast cancer] and the BCMA-targeted ADC belantamab mafodotin for multiple myeloma. Among the kinase inhibitors approved were the HER2 kinase inhibitor tucatinib, together with the RET kinase inhibitors selpercatinib and pralsetinib with indication for RET fusion-positive NSCLC. Lurbinectedin, approved for multiple myeloma, covalently binds to the DNA minor groove. Despite the continued impact of COVID-19, 15 new cancer drugs were approved by the FDA in 2021. The allosteric inhibitor sotorasib targets KRAS-G12C mutated NSCLC, while the novel allosteric HIF-2α inhibitor belzutivan targets von Hippel-Lindau tumours. Dostarlimab, a PD1/PDL1-targeted antibody for endometrial cancer was approved, together with the ADCs loncastuximab teserine, a CD19-targeted ADC for B-cell lymphomas, and tisotumab vedotin, a tissue targeted ADC approved for cervical cancer. The bispecific antibody amivantamab targeting EGFR and MET gained approval for small molecule-resistant NSCLC, the kinase inhibitor mobocertinib selectively inhibits EGFR in NSCLC and the kinase inhibitor asciminib was approved for Philadelphia chromosome-positive CML. Several BCMA-targeted CAR-T cell therapies were also approved in 2021.

Protein kinase inhibitors (PKIs) are clinically significant drugs in the treatment of cancer and inflammatory diseases, with over 535 reported PKs and over 70 PKIs approved by the FDA. Imran et al. have reviewed the USFDA PKI patent approvals for the period 2001 to 31 May 2021 and have provided a comprehensive timeline depicting the PKI approvals, molecular structures, primary targets and approved indications [1]. Availability of generic PKI drugs in the USA market is also discussed together with the development of PKIs with structurally varied scaffolds, chemotypes and pharmacophores. The development of larotrectinib and entrectinib as tissue-agnostic anti-cancer tropomycin receptor kinase (Trk) inhibitors is reviewed by Han [2]. In clinical trials with larotrectinib and entrectinib in patients with a wide range of tumour types with various types of Trk fusion, clinical benefits were observed indicating tumour-agnostic activity. It is concluded that the adoption of the tissue-agnostic approach has accelerated the clinical development of Trk inhibitors.

The simultaneous inhibition of multiple protein kinases targets involved in cancer progression is a possible route to increasing potency and overcoming resistance. Many multi-kinase inhibitors occupy only the hinge and hydrophobic region in the ATP binding site. Mashelkar et al. designed multi-kinase inhibitors that occupy the ribose pocket, along with the hinge and hydrophobic region [3] and identified a novel 4′-thionucleoside with potent anticancer activity and marked inhibition of TRKA, CK1δ, and DYRK1A/1B kinases, with potential for developing anti-cancer drugs. Previtali et al. reported the anti-cancer mechanism of a novel 3,4′-substituted diaryl guanidinium compound that inhibits BRAF through a hypothetical type-III allosteric mechanism [4]. Following a docking study using an active triphosphate-containing BRAF protein, a variety of structural modifications were evaluated in leukaemia, breast, cervical and colorectal carcinoma cell lines with pro-apoptotic effects. A divergent effect on inhibition of MAPK/ERK signalling pathway was demonstrated, confirming that diaryl guanidinium compounds are excellent hit molecules for new anticancer therapies. Elrayess et al. reported a series of thieno[2,3-d][1,2,3]triazine and acetamide derivatives as dual epidermal growth factor receptor (EGFR) and human EGFR-related receptor 2 (HER2) inhibitors targeting non-small cell lung cancer (NSCLC) [5]. The lead compound was cytotoxic at nanomolar levels in the H1299 cell line, with activity against EGFR and HER2 comparable to imatinib and was identified as a promising agent for NSCLC.

Ibrahim et al. investigated the design and synthesis of a series of dual targeting hybrid molecules by combining histone deacetylase (HDAC) inhibition with epidermal growth factor receptor (EGFR-TK) inhibition [6]. The novel hydroxamic acid hybrids were cytotoxic in cancer cell lines, proapoptotic, showed increased expression of caspases 3/8 and Bax and down-regulation in Bcl-2 and inhibition of both EGFR and HDAC1 enzymes. Balbuena-Rebolledo identified several FDA-approved drugs as potential inhibitors of the intracellular domain of epidermal growth factor receptor 1 (EGFR) and human epidermal receptor 2 (HER2) which are important targets for cancer drugs [7]. FDA-approved drugs with similar structures to lapatinib and gefitinib were identified in the DrugBank. Docking and molecular dynamics simulations on the selected compounds identified interactions with the ligand-binding sites of EGFR and HER2, without interaction with residues involved in drug resistance; cytotoxicity was confirmed in breast cancer cell lines. These repurposed compounds may offer possible new anticancer treatments by targeting HER2 and EGFR. 

Sorafenib is an orally administered kinase inhibitor used to treat advanced hepatocellular and renal cell cancer. Inconsistencies in treatment efficacy and tolerability may be attributed to variability in sorafenib exposure over time. Ruanglertboon et al. developed a concentration-guided sorafenib dosing protocol to increase the proportion of patients that achieve a sorafenib C_max_ within the required range by using a model to simulate sorafenib exposure [8]. 

Nagy et al. investigated the inhibition of Bcl-2 as a promising strategy for cancer treatment [9]. Benzimidazole and indole-containing analogues of the Bcl-2 inhibitor obatoclax were designed by introduction of alkylamine or carboxyhydrazine methylene linkers to facilitate improved hydrophobic Bcl-2 binding. Anti-cancer activity was confirmed in MDA-MB-231 (breast cancer) and A549 (lung adenocarcinoma) cells with significantly upregulated expression of pro-apoptotic Bax and caspase-3, -8 and -9, and downregulation of anti-apoptotic Bcl-2. Espadinha et al. reported indole-based tryptophanol-derived polycyclic compounds as activators of the tumour suppressor protein p53, a therapeutic target in many cancers [10]. A novel series of enantiomerically pure tryptophanol-derived small molecules was optimised, and absolute configuration established by X-ray analysis. These compounds target human gastric adenocarcinoma (AGS) cells while mediating apoptosis via increase in caspase 3/7 activity. In vitro stability and metabolic studies identified potent lead compounds for further studies. Stecoza et al. have developed a series of new 2,5-diaryl/heteroaryl-1,3,4-oxadiazoles as novel chemotherapeutic agents [11]. Following evaluation in human colon and breast cancer cell lines, STAT3 and miR-21 are suggested as the most probable targets for these compounds suggesting future studies to improve the anticancer profile and to reduce the toxicological risks. 

The nuclear export receptor exportin-1 (XPO1, CRM1) is a relevant target in haematological malignancies. The XPO1 inhibitor leptomycin B interacts with XPO1 by covalent interaction with Cys528. Gargantilla et al. synthesised a series of chalcones designed to react with XPO1 thiol groups via hetero-Michael addition reactions [12]. Reactions of selected chalcones with GSH demonstrated potential reversible covalent interaction with XPO1 thiols. Good correlation was observed in antiproliferative assays with cancer cell lines and as XPO1 inhibitors. Thymidylate synthase (TS) is an established target in cancer treatment, as it is directly involved in DNA synthesis. Alam et al. developed potential chemotherapeutic hybrid compounds containing 1,2,3-triazole and 1,3,4-oxadiazole heterocycles [13]. Evaluation for inhibition of breast and human colorectal carcinoma demonstrated superior activity to tamoxifen and 5-fluorouracil, with inhibition of thymidylate synthase enzyme superior to pemetrexed. The DNA repair enzyme tyrosyl-DNA-phosphodiesterase 1 (TDP1) acts by removal of TOP1-DNA adducts stabilized by TOP1 inhibitors. The combination of a terpene resin acid backbone with an adamantane fragment as a DNA repair inhibitor was reported by Kolaleva et al. [14]. The linker type and length, diterpene and adamantane moieties were optimised. The synthesized compounds were effective inhibitors of TDP1 while molecular modelling indicated that the TDP1 intermediate (covalent complex of TDP1 with DNA) may be stabilised as observed for topoisomerase−DNA covalent complexes by camptothecins. The highly glycosylated transmembrane mucin (MUC) proteins are over-expressed in different types of cancers and are both promising cancer therapeutic targets and also biomarkers for human cancer. Current efforts to develop MUC1- and MUC16-targeted cancer therapies include antibody-based therapeutics, small molecule inhibitors, vaccines and cell therapies. Lee et al. have comprehensively reviewed the various therapeutic agents targeting mucins which are under different stages of clinical trial for several cancers [15]. 

The synthesis and biochemical evaluation of novel hybrids of the microtubule targeting benzophenone phenstatin and the aromatase triazole inhibitor letrozole are reported by Ana et al. [16]. The compounds demonstrated potency in MCF-7 and MDA-MB-231 breast cancer cells, together with significant G_2_/M phase cell cycle arrest, induction of apoptosis, inhibition of tubulin polymerisation and selective inhibition of aromatase. These hybrids are promising candidates for development as antiproliferative, aromatase inhibitory and microtubule-disrupting agents for breast cancer. The antitumour activity of hybrid compounds based on the structure of combretastatin A-4 and 2,3-diphenyl-2*H*-indazole has been evaluated by Perez-Villanueva et al [17]. Selected hybrid compounds possess significant cytotoxic activity superior to cisplatin against HeLa and SK-LU-1 cells, with similar potency to imatinib against K562 cells, inhibited tubulin polymerisation and induced G_2_/M arrest. Balandis et al. synthesized a series of new imidazole derivatives incorporating a novel benzenesulfonamide moiety [18]. Evaluation against MDA-MB-231 breast cancer and human malignant melanoma (IGR39) cell identified the optimal aryl and imidazole substitution required. A core pharmacophore for the design of anticancer compounds against aggressive and invasive cancers such as malignant melanoma and triple-negative breast cancer was identified. 1,3-Disubstituted derivatives of urea and thiourea are reported to possess antiproliferative properties against various solid and leukaemia tumour cell lines. Strzyga-Lach et al. have developed a series of selective 3-(trifluoromethyl)phenylthiourea analogues with selective cytotoxic effects against human colon, prostate and leukaemia cell lines [19]. The most potent compounds showed pro-apoptotic activity and inhibited release of the cytokine IL-6 in the colon SW480 and SW620 cells lines. The anticancer effects of xanthones may be attributed to caspase activation, DNA cross-linking, inhibition of kinases and topoisomerase. Recent advances in the discovery of xanthone derivatives with anticancer activity, both isolated from natural sources and synthetic examples are reviewed by Kurniawan et al., together with potential further developments of active, selective and efficient anticancer drugs based on xanthone derivatives [20]. 

Toxicity and drug resistance remains a challenging issue in cancer drug development. The type II topoisomerase inhibitor mitoxantrone (MTX) is used in to treat several cancers and refractory multiple sclerosis. Reis-Mendes et al. investigated the cardiotoxicity of MTX to determine if inflammation or oxidative stress-related pathways are involved [21]. Histopathology results indicated that MTX caused cardiotoxicity while inflammation may be an important trigger to MTX-induced cardiotoxicity in adult mice, with increased expression of NF-κB p65, NF-κB p52 and TNF-α with decrease in IL-6. The widely used DNA alkylating agent cyclophosphamide (CPX) causes toxic effects in the urogenital system. Merwid-Ląd examined the effect of morin-5′-sulfonic acid sodium salt (NaMSA) on CPX-induced urogenital toxicity in rats by histological evaluation, morphological changes and relative decrease in sperm count [22]. Co-administration of NaMSA reversed most of the morphological changes and may attenuate CPX-induced histological changes in the urogenital tract. The nephroprotective effects of the beta-adrenergic antagonist carvedilol on CPX-induced urotoxicity was also examined [23]. When co-administered with mesna, carvedilol improved kidney function and reversed histological abnormalities in bladders, presumably via antioxidant and anti-inflammatory effects. Multidrug-Resistant (MDR) cancers modulate chemotherapeutic efficacy through drug efflux. Pulukuri et al. investigated P-glycoprotein 1 mediated efflux of Toll-Like Receptor (TLR 7/8) agonist immunotherapies [24]. The imidazoquinoline TLR agonists imiquimod, resiquimod and gardiquimod are P-gp substrates. Imidazoquinoline efflux occurs through P-gp and is enhanced for imiquimod due to acquired drug resistance. This enhancement could be beneficial for modulating the activity of tumour-infiltrating immune cells in local proximity to cancer cells

The research presented in this Special Issue contains contributions which are focussed on cancer drug discovery together with important reviews in specific areas of cancer therapeutics. This Special Issue highlights both the challenges and opportunities in the discovery and development of both novel small-molecule cancer drugs and applications of innovative cancer therapies and demonstrates the direction and potential for future research in these areas. 

## Data Availability

Data is contained in the article.
